# Coconut Carbon Dots: Progressive Large-Scale Synthesis, Detailed Biological Activities and Smart Sensing Aptitudes towards Tyrosine

**DOI:** 10.3390/nano12010162

**Published:** 2022-01-03

**Authors:** Pooja Chauhan, Deepa Mundekkad, Amitava Mukherjee, Savita Chaudhary, Ahmad Umar, Sotirios Baskoutas

**Affiliations:** 1 Centre of Advanced Studies in Chemistry, Department of Chemistry, Panjab University, Chandigarh 160014, India; pujachauhan.05@gmail.com; 2 Centre for Nanobiotechnology, Vellore Institute of Technology, Vellore 632014, India; deepamundekkad@gmail.com (D.M.); amit.mookerjea@gmail.com (A.M.); 3 Department of Chemistry, College of Science and Arts, Najran University, Najran 11001, Saudi Arabia; 4 Promising Centre for Sensors and Electronic Devices (PCSED), Najran University, Najran 11001, Saudi Arabia; 5 Department of Materials Science, University of Patras, 265 04 Patras, Greece

**Keywords:** carbon dots, amino acids, biocompatible, sensing, glutamic acid

## Abstract

In the recent era, carbon dots (C-dots) have been extensively considered as a potential tool in drug delivery analysis. However, there have been fewer reports in the literature on their application in the sensing of amino acids. As part of our ongoing research on coconut-husk-derived C-dots, we synthesized C-dots under different temperature conditions and utilized them in the field of amino acid sensing and found them to be highly selective and sensitive towards tyrosine. The detailed characterization of the prepared C-dots was carried out. The developed C-dots exhibit good values of quantum yield. BSA, HSA and glutamic acid were utilized to explore the binding efficiency of C-dots with biologically active components. Hemolysis, blood clotting index activity and cell viability assays using the prepared C-dots were evaluated and they were found to be biocompatible. Therefore, the C-dots described in this work have high potential to be utilized in the field of amino acid sensing, especially L-tyrosine. The limit of detection and the binding constant for the developed C-dots in the presence of tyrosine were found to be 0.96 nM and 296.38 nM^−1^, respectively. The efficiency of the developed C-dots was also investigated in the presence of various other amino acids and different water mediums in order to enhance the working scope of the developed sensors.

## 1. Introduction

The sensing of 4-hydroxyphenylalanine (or) L-tyrosine is highly relevant to research in the analytical, material and life sciences [1,2]. As one of the important amino acids, L-tyrosine is meaningfully used by cells for formulating proteins in living systems [3]. Correct nitrogen balancing in the body is controlled by L-tyrosine [4]. The effective translation of L-tyrosine into melamine via skin cells has a defensive impact on the body due to the unfavorable effects of ultraviolent radiation [5,6]. Any kind of abnormality in the concentration levels of L-tyrosine can lead to several disorders, such as hypo- and hyper-thyroidism, in living beings [7]. In addition, dementia, depression, Parkinson’s disease, tyrosinemia and disfunction of the kidney and liver have also been reported due to unbalanced concentrations of L-tyrosine in living systems [8,9]. Additionally, increased concentrations of L-tyrosine in the body has caused atherosclerosis, joint pains and heartburn in some cases [10]. Therefore, an economically efficient means of analyzing the levels of L-tyrosine is highly needed to ensure the wellbeing of living systems. To date, various sophisticated analytical methods, including chromatography, electrophoresis, mass spectral analysis and electrochemical methods, have been employed for the recognition of L-tyrosine [11,12]. Conversely, the costly instrumentation, time-consuming sample processing and the need for skilled professional experts have restricted the widespread use of these available methodologies in the large-scale sensing of L-tyrosine. However, in comparison to the existing methodologies, fluorescence-based methods have provided a cost-effective, selective and sensitive alternative method for the detection of low concentrations of L-tyrosine. The ease of calibration of the emission intensity of existing fluorophores, as per the available concentration of analytes, has enhanced the working scope of this method in the field of sensing [13,14,15]. To date, several single-intensity-dependent fluorescence probes have been utilized for the detection of L-tyrosine. For instance, Zribi et al. developed the enzyme-free method for the detection of L-tyrosine using the application of molybdenum disulphide nanosheets (2D-MoS_2_) [16]. The catalytic nanomaterials were prepared using the liquid phase exfoliation method and possessed a maximum sensitivity and detection limit of 1580 μAmM^−1^ cm^−2^ and 0.5 μM, respectively, for L-tyrosine. Furthermore, Vyas et al. used the application of a calix[4]arene-based compound for the selective sensing of L-tyrosine, with an LOD of 1.2 ppm in aqueous media [14]. However, the formation of sensory probes is quite complex and this had made the process less feasible for the detection of analytes. In addition, the concentration changes of the probe and light scattering by the media further affect the sensing ability of the fluorophore.

In this regard, carbon dots have shown significant potential in providing a ratiometric fluorescence response towards low concentrations of analytes in aqueous media [17,18,19,20]. Being a metal free-fluorophore, carbon dots are considered to be one of the economically viable materials with high water solubility and photo-stability. The highly biocompatible nature of carbon dots has made them a useful material in biomedicines as a way of providing effective alternatives for drug delivery in chemotherapy [21,22,23]. The advanced optical and luminescence properties of carbon dots (C-dots) have demonstrated impressive potency in photocatalysis, chemosensing, light emitting diodes (LEDs) and electrochemical sensing [24,25,26]. In addition, carbon dots have been considered as a novel fluorophore for the bioimaging of in vivo living cells and tissues [27,28,29].

Inspired by the promising scope of applications of carbon dots, we aimed to fabricate a highly selective and sensitive fluorescent probe with carbon dots for the detection of L-tyrosine. The carbon dots were synthesized through the single-step thermal calcination of coconut husk as the precursor source and referred to as “C-dots”. As one of the most important oilseed crops, coconut production has been significantly rising annually [30,31,32]. In India, around 14.19 million metric tons of coconuts were produced in the year 2020. High coconut production has been required to fulfil the rising demands of various food and cosmetic industries [33,34]. As a result, the management of waste coconut husks has aroused wide concern among researchers. However, several methods are available for reusing this waste in the manufacturing of ropes and as a feedstock for the production of charcoal. However, the low heating rate due to the presence of a high water content and the presence of sulfur causes a foul smell and other deterioration-related issues. Additionally, the risk of fire during the transportation of fibrous powder from coconut waste has restricted their consumption to a great extent [35,36]. The higher operational cost associated with the use of agricultural waste in power plants has further made this less favorable in thermal stations. Therefore, the development of effective methods for reusing coconut waste is a highly challenging research area confronting researchers [30,37].

The current work offers an effective and alternative means to convert coconut husk waste into highly fluorescent C-dots for label-free fluorescent probes targeting L-tyrosine. The current work fills the gap in our knowledge on the available experimental research involved in converting coconut husks into C-dots. The information gaps regarding the effect of thermal calcination temperatures on the conversion of coconut husks to C-dots and its consequences in regard to the size, shape, morphology, optical and luminescence properties during the formation of C-dots have been thoroughly investigated in this work. Additionally, the binding efficiency of formed C-dots with BSA, HSA and glutamic acid has been investigated to verify the biocompatibility of the formed particles. The hemolysis and blood clotting index activity of the prepared C-dots were evaluated and their cell viability was examined through the MTT assay. The current work offers the possibility to future researchers of converting waste biomass to highly advanced, biocompatible, and selective fluorophores. Furthermore, the prepared C-dots provide a “turn-off” sensor probe and have the potential for use in intracellular imaging for the detection of L-tyrosine by using highly biocompatible and non-lethal C-dots.

## 2. Experimental Section

### 2.1. Reagents and Chemicals

The lyophilized powder form of bioreagent-grade bovine serum albumin (BSA) and human serum albumin (HSA) were procured from Sigma-Aldrich, Bangalore, India, with purity <98%. Phosphate buffer with pH 7.4 was prepared in a laboratory by mixing sodium phosphate dibasic and sodium phosphate monobasic. Both sodium phosphate salts were purchased in anhydrous form with 99% purity from Sigma-Aldrich. Twenty amino acids (aspartic acid, serine, alanine, glycine, leucine, isoleucine, proline, valine, phenylalanine, tyrosine, tryptophan, histidine, glutamic acid, cysteine, asparagine, glutamine, lysine, arginine, methionine, and threonine) were purchased from Sigma-Aldrich, Bangalore, India with 99% purity. Inorganic anions (SO_4_^2−^, Cl^−^, SO_3_^2−^, NO_3_^2−^, NO_2_^−^, SO_4_^2−^, CO_3_^2−^, HCO_4_^−^, and S_2_O_8_^2−^) of barium salt were purchased from Avra chemicals, Hyderabad, India with 98% purity. Deionized water was utilized during all experimental procedures for the preparation of samples. 3-(4,5-dimethylthiazol-2-yl)-2,5-diphenyl tetrazolium bromide (MTT) and 2’,7’-dichlorodihydrofluorescein diacetate (DCFH-DA) were purchased from Sigma-Aldrich, Bangalore, India. Murine macrophage cell lines (RAW 264.7) were purchased from the National Centre for Cell Science (NCCS), Pune, India. The cells were maintained in DMEM, supplemented with 10% fetal bovine serum (FBS), 100 mg/L streptomycin, and 100 IU/mL penicillin, at 37 °C in a 5% CO_2_ incubator. All the samples were used without any further purification.

### 2.2. Fabrication of Coconut-Derived C-Dots via Thermal Calcination

**Figure 1:**
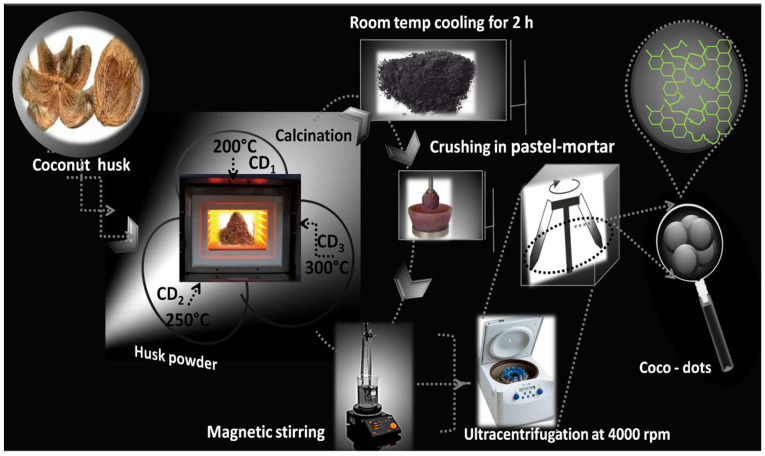
Schematic illustration showing the synthesis of C-dots using the thermal calcination treatment of coconut coir as a precursor material.

The fabrication of C-dots was carried out through the thermal calcination of waste coconut coir as a precursor material. First, coconut coir was cut into small pieces utilizing sterilized scissors. Afterwards, 50 g of small pieces were shifted to a silica crucible and allowed to set in a preheated muffle furnace at three different temperatures i.e., 200 °C, 250 °C, and 300 °C for 2 h to obtain CD_1_, CD_2_, and CD_3_, respectively. The resultant grayish-black powder was allowed to cool at room temperature, followed by fine crushing using electric pastel mortar. Afterwards, the large aggregates of the obtained powder were removed by sieving it through 0.22 mm mesh. The resultant fine powder was dispersed in water and allowed to stir at room temperature for 20 h. The supernatant was collected from the prepared suspension and centrifuged at 4000× *g* for 5 min. The obtained suspension was filtered through a 0.22 µM filter membrane and a further dialysis bag of 1000 MWCO was utilized to extract out the impurities. The attained solution was heated at 102 °C to obtain the powdered form of C-dots [17]. The prepared sample was further kept at 2 °C for subsequent analytical sampling (Figure 1). The detailed flowchart for the fabrication of C-dots is represented in Scheme S1.

### 2.3. Characterizations

The thermal insulating environment was provided by a AICIL muffle furnace during the synthesis of C-dots. The purification of C-dots was performed on a Remi R-24 centrifuge at 5000× *g* and on a power sonic 405 Sonicator. The homogenous suspension of C-dots was prepared utilizing an IKA, C-MAG HS7 magnetic stirrer (Karnataka, India) with a heating plate. A Jasco V-750 UV-vis. spectrophotometer (Easton, MD, USA) was used to evaluate the absorption behavior of C-dots in a quartz cuvette in a fixed path length of 1.0 cm and a working range of 200–800 nm. Furthermore, the emission profile was assessed by employing a Perkin Elmer LS 55 spectrofluorometric instrument (Waltham, MA, USA) in a working range of 200–800 nm and average scan range of 500 nm. The hydrodynamic size distribution and polydispersity index of the formed C-dots were checked on a MALVERN ZEN 1690 dynamic light scattering (DLS) instrument (Malvern, UK). The existence of different kinds of functional groups over the exterior surfaces of the formed C-dots were checked using Perkin Elmer (RXI) Fourier transform infrared (FTIR) spectroscopy (Waltham, MA, USA) in a working range from 500 to 4000 cm^−1^. The purity, morphological changes, and elemental mapping of the formed C-dots were tested on a field emission-scanning electron microscopy (FE-SEM) instrument (JEOL JSM 7500F, Tokoy, Japan) with an energy dispersive X-ray spectroscopy (EDX) unit at 20 KeV. Simultaneously, the direct imaging and their distribution profile was assessed on a high-resolution transmission electron microscope (HRTEM) H-7500 from Hitachi (Tokoy, Japan) at 100 kV. The crystalline behavior of the developed C-dots was verified using a Panalytical X’Pert Pro powder X-ray diffractometer (XRD, Davis, CA, USA) with monochromatic CuKα radiation between angles of 5°–90° and an average scan range of 2 deg/min. The crystalline size was calculated by using the Debye Scherrer formula D = Kλ/βCosθ, where K corresponds to a dimensionless shape factor of which the value is taken as unity (0.94), λ refers to the X-ray wavelength with a value of 0.154 nm, β corresponds to the full width at half maxima (FWHM) intensity and θ corresponds to Bragg’s angle calculated based on XRD data. The effect of pH (acidic and basic conditions) on the surface properties of the developed C-dots were investigated using a Mettler Toledo digital pH meter. The thermal characteristic behavior of the formed C-dots was inspected using an SDT Q-600 thermo-gravimetric analytical differential scanning calorimeter (TGA-DSC) instrument under nitrogen atmosphere in a working range of 0–1000 °C and a Mettler Toledo Q20 instrument (Columbus, OH, USA) in a working range of 0–350 °C, respectively. The zeta-potential values of pure C-dots and in the presence of different types of biological components were inspected on an Anton Paar Litesizer 500 instrument (Graz, Austria) with Omega measurement cells.

### 2.4. Quantum Yield

The quantum yield (Φ) value is considered one of the significant parameters in the case of active fluorescent nanodots. It is defined as the number of photons emitted, divided by the number of photons absorbed by the system. Its value directly depends upon the chemical structure and environment of the developed fluorescent system. During fluorescence spectroscopy, it has been observed that after molecules absorb photons, they do not always emit the same number of photons because of non-radiative relaxation from the excited state to the ground state, which leads to a quantum yield value <1 (100%). Here, tyrosine and tryptophan were utilized as reference standards (Φ = 0.14 and 0.13) for the calculation of the quantum yield of prepared C-dots under different temperature conditions. For this analysis, an aqueous solution of each respective amino acid was prepared and its absorption and emission analyses were carried out. The formula mentioned below was used for the calculation of quantum yield:

(1) $$\phi_{x}=\phi_{r}{\left(\frac{I_{x}}{I_{r}}\right)}_{}{\left(\frac{\eta_{x}^{2}}{\eta_{r}^{2}}\right)}_{}{\left(\frac{A_{r}}{A_{x}}\right)}^{\;}$$

where x and R refer to the C-dot sample and reference material, respectively; I corresponds to the integrated emission intensity; η corresponds to the refractive index of the material; and A corresponds to the absorbance values determined based on the absorption spectrum [38,39,40,41,42].

### 2.5. Physiological Activity of C-Dots

#### 2.5.1. Time Studies

To investigate the photochemical stability of the prepared C-dots in aqueous medium, a time replication study was carried out. For the respective study, a fixed concentration (2 mg in 5 mL) of C-dots was kept for different intervals of time and their fluorescence spectra were recorded in working range of 200–800 nm.

#### 2.5.2. Influence of pH

The influence of pH on the synthesized C-dots was also tested by recording the fluorescence behavior of the formed samples with different pH values at room temperature. For this analysis, six different pH solutions (2.6, 4.8, 5.8, 7.6, 10.1, and 13.1) of C-dots were prepared using dilute HCl and NaOH solutions and their fluorescence spectra were recorded in the range of 200 to 800 nm.

#### 2.5.3. Salt Effect Analysis

The respective salt effect activity was analyzed to obtain information about the ionic strength of the C-dots in the presence of different ions. Their emission behavior in the presence of different salts was assessed using fluorescence spectrophotometric measurements. Solutions of NaCl and KCl with different concentration ranges of 0 mM, 100 mM, 250 mM, and 500 mM were prepared in deionized water and their emission behavior was tested using fluorescence spectroscopy in the range of 200 to 800 nm.

### 2.6. Biological Activity of C-Dots

#### 2.6.1. Binding Mechanism

The UV-vis. spectroscopic measurements were carried out in order to investigate the binding capacity of the formed C-dots with BSA, HSA and glutamic acid. The 0.2 M phosphate buffer solution was chosen as the reference sample during UV-vis analysis. The sample of C-dots with different concentrations ranging from 2 mg to 9 mg in 5 mL were prepared in 0.2 M phosphate buffer solution (pH = 7.4) for the analysis. During the measurements, the concentration of BSA was fixed at 1.25 mg per mL of solution. Similarly, the concentration of HSA protein and glutamic acid was kept at 1.0 mg per ml of solution. The stock solutions of all the biological components were prepared in 0.2 M phosphate buffer. The electrical charge and stability of developed system was checked using zeta potential measurements [43].

#### 2.6.2. Hemolysis and Blood Clotting Index Value in the Presence of C-Dots

The hemolysis assay provides information about the damage to erythrocytes in the presence of external moieties. For this analysis, fresh blood samples from a volunteer were procured in EDTA-coated vials to explore the hemolysis and blood clotting index of the developed C-dots. The reduction of the concentration in freshly acquired blood samples (human erythrocytes) was measured using 1% saline solution in the presence of different concentration levels (2 to 9 mg/mL) of C-dots. Known and equal volumes of C-dots mixed with 0.2 mL of diluted blood samples were prepared in suspension form and allowed to incubate at 37 °C for 50 min. The respective solutions were centrifuged using physiological saline solution for 4 min at 1000× *g* and the supernatant was collected and their absorbance values were measured at 540 nm to evaluate the released amount of hemoglobin. Further, to test the blood clotting index of the prepared C-dots, 0.2 M CaCl_2_ solution was gradually assorted with the freshly collected blood samples to prevent the rupturing of blood plasma. Subsequently, 0.5 mL of differently concentrated solutions of C-dots was allowed to mix with the fresh blood suspension and incubated at room temperature for 15 min. Afterwards, 3 mL deionized water was added to the above prepared samples and centrifuged at 1000× *g* for 4 min and supernatant collected from the respective solutions was diluted further by adding deionized water and was again incubated at 37 °C for 10 min. The absorbance value for each sample was recorded at 542 nm. The values of the blood clotting index and hemolysis ratio were calculated using the following equations:

Blood clotting index = (OD_sample_/OD_blood_) × 100 (2)

Hemolysis ration = (OD_sample_ − OD_negative_)/(OD_positive_ − OD_negative_) (3)

Here, OD corresponds to the optical density (absorbance) values, subscript positive indicates a sample diluted with water, and negative refers to a blood sample diluted with the saline solution [44,45].

#### 2.6.3. MTT Assay

The reduction of yellow 3-(4,5-dimethylthiazol- 2-yl)-2,5-diphenyl tetrazolium bromide (MTT) by mitochondrial succinate dehydrogenase to an insoluble purple-colored formazan product is the basis of the MTT cytotoxicity assay [46]. The formazan crystals formed can be solubilized with an organic solvent such as dimethylsulphoxide and the color developed can be measured colorimetrically at 570 nm. The cytotoxic effect of the formed C-dots on the cells was measured by means of an MTT colorimetric assay, according to the protocols in [46] with slight modifications [46]. Raw macrophage cell lines in their exponential growth phase, at a density of 1 × 10^4^ cells/mL, were seeded into a 96-well culture plate, and left overnight for incubation. After attachment, the cells were treated with different concentrations of the C-dots and incubated for another 24 h. The media containing C-dots were removed and MTT (25 µL of 5 mg/mL) was added to the wells and kept for 4 h at 37 °C in the incubator. After removing the supernatant, the formazan crystals formed were dissolved in 100 µL DMSO. The absorbance of the resultant solution was read at 570 nm using a microplate reader (BioRad). The percentage of viability of the cells was calculated as

% viability = (absorbance of treated cells/absorbance of control cells) × 100(4)

#### 2.6.4. ROS Detection Using DCFH-DA

Intracellular ROS generation/scavenging after treating the cells with C-dots was measured via the 2′,7′-dichlorodihydrofluorescein diacetate (DCFH-DA) fluorescent staining assay. RAW macrophages grown to confluency were treated with different concentrations of C-dots at 37 °C for 24 h in an incubator. The media containing C-dots were removed and 500 μL of DCFH (10 μM) solution (prepared immediately before the experiment) was added to the wells. The cells were incubated for 30 min. DCFH-DA is a non-fluorescent dye and shows fluorescence in its reduced state when the acetate groups are removed using intracellular esterase to produce green fluorescence [47]. The green fluorescence emitted by the cells, which is a visible sign of the presence of intracellular ROS, was read using a fluorescent spectroscope (Agilent Technologies, Santa Clara, CA, USA). The fluorescent intensity increases/decreases depending on the ROS inducing/scavenging capacity of the C-dots. The cells without any treatment served as control cells. Non-parametric one-way ANOVA was carried out using the data, and Dunnett’s multiple comparisons test results were employed to test significance at *p* < 0.05.

### 2.7. Response Behavior of C-Dots towards Amino Acids

#### 2.7.1. Sensing Procedure

One-micromole solutions with concentrations of the chosen amino acids were prepared by sonicating the prepared samples for 30 min. Around 2 mg of powdered C-dots was suspended in 5 mL of deionized water and allowed to sonicate, forming a homogenous dispersion, then was left undisturbed for 30 min at room temperature under optimal conditions. Afterwards, a 1 mL solution of the prepared amino acid solution was allowed to mix with an equal volume of C-dots. The fluorescence spectrum for each prepared sample was recorded in a working range of 200 to 800 nm. Upon interpreting the results, it was observed that the formed C-dots were quite selective and sensitive for tyrosine.

#### 2.7.2. Concentration-Dependent Study of Tyrosine

After observing the selectivity of tyrosine of the formed C-dots, studies of their concentration variations were performed in terms of a titration scheme using fluorescence spectro-photometric measurements. For analysis, the concentration of tyrosine was varied from 100 nM to 1 nM and their detection limit values were calculated.

#### 2.7.3. Calculation of Detection Limit and Quantitation Limit

The values of the detection limit (σ) and quantitation limit (β) were calculated using a Stern–Volmer plot (F/F_0_ vs. concentration) by applying the formula:

σ = 3S.D/Slope(5)

β = 10S.D/Slope(6)

where S.D refers to the standard deviation of the C-dots, and the slope was evaluated by plotting the linear calibration curve of the emission intensity of C-dots in the presence of amino acids and at different concentrations of amino acids used [25].

#### 2.7.4. Calculation of Binding Constant

A Benesi–Hilderbrand plot (1/F − F_0_ vs. conc.) was drawn between emission intensity and the reciprocal of the tyrosine concentration in order to estimate the binding efficiency and stoichiometry of the C-dots with tyrosine amino acid using the following equation:

Binding constant (B.C) = Intercept/Slope(7)

#### 2.7.5. Interference Studies

The sensitivity of the developed sensor was further tested by investigating the interfering influence of different types of molecules and ions using fluorescence spectroscopy. For the respective studies, the fluorescence measurements were carried out for 1 mL of an aqueous solution of C-dots in tyrosine solution in the presence of other amino acids.

#### 2.7.6. Analysis of Real Water Samples

The practical scope of developed tyrosine sensor was assessed using C-dots in real water samples (tap water, well water, and rainwater) through fluorescence spectroscopy. For the respective activity, 1 mL of the C-dot suspension was mixed with 1 mL of tyrosine. The sample preparation of Tyrosine with different concentration levels (1, 2, 3, 4, and 5 nM) was carried out in different real wastewater media. The emission profiles of the respective samples were analyzed in a working range of 200–800 nm. All the experimentation were carried out in triplicate in order to scrutinize the applicability of the respective system [21,25].

## 3. Results and Discussion

### 3.1. Characterization of C-Dots

#### 3.1.1. Optical, Emission, Surface, Structural, and Morphological Characteristics of C-Dots

**Figure 2:**
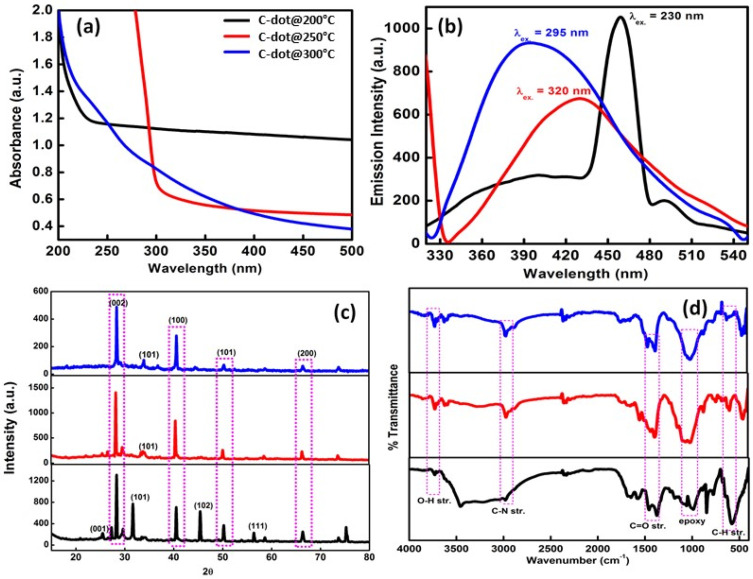
(**a**) UV-vis. absorption spectrum, (**b**) fluorescence excitation spectrum, (**c**) XRD and FTIR spectra of the formed C-dots under three different temperature conditions, i.e., 200 °C, 250 °C, and 300 °C. (**d**) the nature of the different types of functional groups on the surface of the C-dots was evaluated through FTIR analysis.

The optical absorption behavior of the developed C-dots at three different temperatures was clearly visualized in the UV-vis spectroscopic studies at a fixed path length (1.0 cm) in the range of 200–500 nm. The absorbance band at 210 nm in the case of CD_1_ was associated with the π-π^*^ transition in the C=C bond present as the structural unit in the C-dots (Figure 2a). In the case of CD_2_, the absorbance band due to the n-π^*^ transition of the C=O bond was observed at 298 nm. However, CD_3_ particles formed via thermal calcination at 300 °C displayed the existence of two absorbance bands at 226 and 284 nm, respectively. These two peaks were mainly aroused due to the existence of n-π^*^ and π-π^*^ transitions in CD_3_ particles. These n-π^*^ transitions generally corresponded to the C=N bond in the formed C-dots, whereas, the π-π^*^ transition was aroused due to the existence of aromatic sp^2^ hybridization in C-dots, along with the C=O (carboxyl) and O-H (hydroxyl) functionality over the exterior surface of the formed particles. In addition, variation in the calcination temperature during the fabrication of the C-dots produced significant shifts in the absorption peaks. This behavioral variation can be explained due to the enhancement in the local refractive index of the surroundings of the formed C-dots. In addition, fluorescence spectroscopy was used to explore the emission behavior of the formed C-dots in the range of 200–800 nm. They display a bright fluorescent blue color under UV illumination. The quantum yield values CD_1_, CD_2_, and CD_3_ were calculated respectively (λ_ex_. = 230, 320, and 295 nm for CD_1_, CD_2_, and CD_3_, respectively) utilizing tyrosine and tryptophan standard dye (λ_ex._ = 280 nm) as a reference material (Table S1). This was mainly due to the modification in the inter-particle distances among particles. The excitation-dependent emission profile of the developed C-dots was also evaluated through fluorescence spectroscopy in the region of 200–600 nm with a fixed bandwidth of 10 nm (Figure S1). The C-dots developed at 300 °C possessed the maximum emission behavior out of the three C-dots. The prepared C-dots exhibited maximum excitation wavelengths at 230, 320, and 295 nm with corresponding emission peaks at 402, 432, and 400 nm for CD_1_ to CD_3_, respectively (Figure 2b). The crystalline size of the developed C-dots was assessed using X-ray diffraction analysis (Figure 2c). In the case of CD_1_, the diffracting plane was observed at 2θ = 25.41°, 28.28°, 40.48°. 45.44°, 50.33°, 56.42°, and 66.48°, corresponding to (001), (002), (100), (100), (102), (101), (110), and (200). In the case of CD_2_ and CD_3_, the diffracting plane was observed at 2θ = 28.26°, 33.83°, 40.45°, 50.36°, and 66.43°, corresponding to (002), (111), (100), (101), and (200). The calculated crystalline sizes of CD_1_, CD_2_ and CD_3_ were 90.43, 62.91, and 51.67 nm, and the dislocation density values were 12.22, 25.26, and 37.45 (10^−5^) nm^−1^, respectively. From these results, it can be concluded that the developed C-dots were completely synthesized in the nanometric range and displayed the well-developed diffraction peaks of carbon dots. The nature of the different types of functional groups on the surface of the C-dots was evaluated through FTIR analysis (Figure 2d). In case of all three types of C-dots formed, the major peak was obtained at 3608 cm^−1^, corresponding to the strong O-H stretching vibration, especially hydrogen bonding, taking place in the system. The peak at 2974 cm^−1^ corresponded to C-N and C-H stretching vibration [17,21]. C=C and C=O stretching vibration was observed at 1378 and 1452 cm^−1^, respectively. The peaks at 994 and 852 cm^−1^ corresponded to the epoxy group and =C-H stretching bands over the surfaces of particles. Some additional observations, such as the presence of some kind of noise and the absence of the proper orientations of peaks in the case of CD_1_ and CD_2_, suggested that the particles were not fully developed. However, the particles formed at 300 °C showed well-orientated peaks. Additionally, the XRD and FTIR spectra of the developed C-dots closely resembles cellulose dominance, which might be due to the cellulose-like material and structure of the coconut husk waste material used as starting precursor for the preparation of the C-dots. Moreover, this coconut husk waste can be utilized as a cellulose material due to its porous, amorphous, and water-resistant nature.

**Figure 3:**
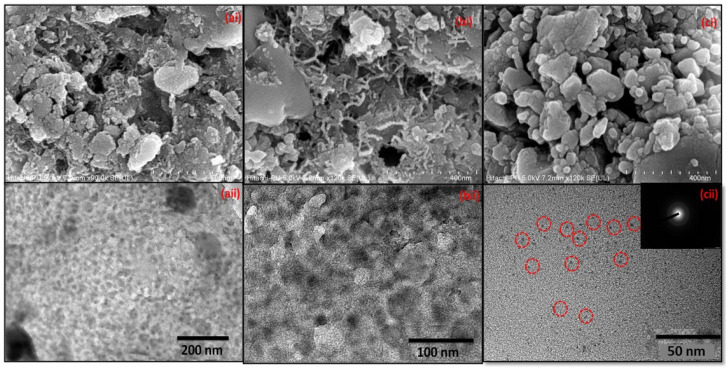
(**ai**–**ci**) FESEM and (**aii**–**cii**) HRTEM images of the C-dots formed under three different temperature conditions, i.e., 200 °C, 250 °C, and 300 °C.

FESEM and HRTEM techniques were utilized to analyze the morphological and topographical symmetry of the developed C-dots. The FESEM analysis of the dried powdered C-dots was carried out to evaluate their morphological behavior in their dried form. Based on the FESEM images (Figure 3ai–ci), it can be seen that the developed C-dots were somewhat aggregated in the case of CD_1_ and CD_2_, with average sizes of 42 and 28 nm. However, the spherical morphology of CD_3_, with an average size range of 12 nm, is clearly visible in the FESEM images (Figure 3ci). In addition, the distribution of particles was found to be homogenous in nature, with an equal distribution of masses in the nano-range and the formed particles were interconnected with each other. Additionally, based on the HRTEM images, it can be inferred that the prepared C-dots were spherical in nature, with average sizes of 50, 36, and 8 nm (Figure 3aii–cii). The SAED pattern in case of CD_3_ showed the formation of proper crystalline C-dots with lattice fringes with average values of 0.25 nm (Figure 3cii). These lattice fringes corresponded to the (100) plane of carbon in the formed particles. The corresponding size distribution profile of the developed C-dots was evaluated using ImageJ software (Figure S2). Furthermore, to verify the purity of the developed C-dots, the respective EDX analysis was carried out (Figure S3). Based on the EDX spectrum, it was observed that the developed C-dots showed the presence of carbon, oxygen, and nitrogen, which suggested the interruption of any other elements in the system. The percentages of carbon, oxygen, and nitrogen were 50%, 48%, and 2%. It can thus be inferred that the prepared C-dots were highly pure in nature. Based on the obtained results, the C-dots synthesized at 300 °C (CD_3_) were considered to be the most appropriate for carrying out further analysis due to their small size, better thermal properties, and excellent absorption and emission properties. They were further utilized in the application of biological component sensing.

#### 3.1.2. Thermal Properties of C-Dots

The thermal stability of the formed particles was assessed using TGA analysis of the formed C-dots. A typical thermo-gravimetric graph showed three major weight losses (Figure S4). In CD_1_, weight losses of 17.69%, 29.48%, and 58.79% were observed at 195 °C, 598 °C, and 985 °C, respectively. In the case of CD_2_, weight losses of 8.94%, 15.54%, and 52.64% occurred at 198 °C, 595 °C, and 995 °C. However, for CD_3_, 17.69%, 18.28%, and 70.92% weight losses were observed at 192 °C, 593 °C, and 987 °C, respectively. The first weight loss was mainly due to the evaporation of water molecules and the moisture content in the sample. The second weight loss corresponded to the evaporation of pyro gases (mostly consisting of carbon dioxide, carbon monoxide, and methane) present on the surfaces of the C-dots. The third weight loss corresponded to the evaporation of different functional groups (carbonyl, hydroxyl, and amine groups) present on the exterior cores of the C-dots. Further DSC analysis was carried out for the developed C-dots to explore their exothermic and endothermic behavior in the form of a heat release. The value of the onset temperature (T_o_), glass transition temperature (T_g_), melting temperature (T_m_), and heat of enthalpy and entropy was calculated for all the prepared types of C-dots (Table S2). Upon interpretation of the results, it was observed that the developed C-dots possessed exothermic behavior.

### 3.2. Physiological Activity of C-Dots

**Figure 4:**
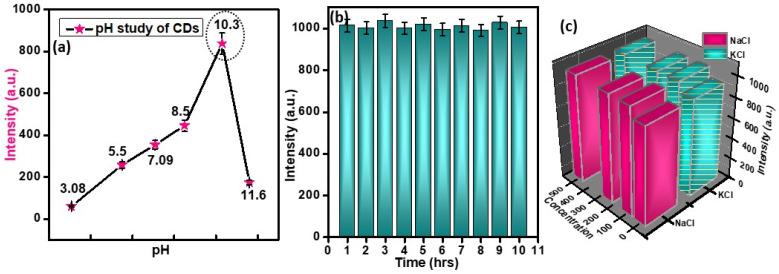
Effect of (**a**) pH, (**b**) time, and (**c**) ionic strength (salts of NaCl, KCl) on the emission behavior of C-dots prepared at 300 °C (with <5% error bars calculated after three measurements for effectiveness and uncertainty).

In order to assess the stability of the formed particles under different physiological conditions, the emission profiles of the highly stable and well-formed C-dots (i.e., CD_3_) were analyzed under different pH, ionic strength, and time conditions. For this analysis, the emission intensity of the formed C-dots was investigated by varying the pH values from 2 to 13 (Figure 4a). Upon interpretation of the data, it was found that the emission intensity for all the formed C-dots showed linear increments on changing the pH from an acidic to an alkaline medium. This behavior continued up to pH values equal to 10 at room temperature. On further raising the pH from 10 to 12, we observed a decrease in the emission intensity of the prepared C-dots. This behavioral change occurred mainly due to the agglomeration tendency of the C-dots in highly basic medium. The obtained results were further verified via absorption spectroscopy, which suggested that the absorption behavior and optical density values did not change much with change in medium of the solution (Figure S5). It can thus be inferred that the formed particles are quite stable in a basic medium up to pH = 10. Simultaneously, variations in the emission intensity were also observed as a function of time in order to test their steadiness and photostability (Figure 4a). We observed that changes of approximately ± 2% in the emission intensity in a span of 11 h of investigation, which pointed towards the high stability of the formed particles (Figure 4b) under exposure to normal visible light. This slight variation in emission intensity occurred due to changes in atmospheric temperature and environmental conditions. The corresponding effect of the ionic strength of different salts on the emission intensity of the prepared particles was assessed to verify the stability of the formed particles (Figure 4c). Three different concentrations were used (100, 250, and 500 mM) to verify the effect of salt stress. It was observed that in the presence of NaCl and KCl, the emission intensity of the C-dots increased rapidly. However, in the presence of a 100 mM concentration of electrolytes, the external surface of the C-dots was completely covered by chloride ions via hydrogen bonding. Therefore, the further addition of electrolytes did not produce any change in the emission profiles of the C-dots. This physiological activity of C-dots well supports the stability of the formed particles in different environmental conditions and thus enhances their potential application as an effective photo-luminescent agent.

### 3.3. Biological Activity of C-Dots

#### 3.3.1. Binding Mechanism

**Figure 5:**
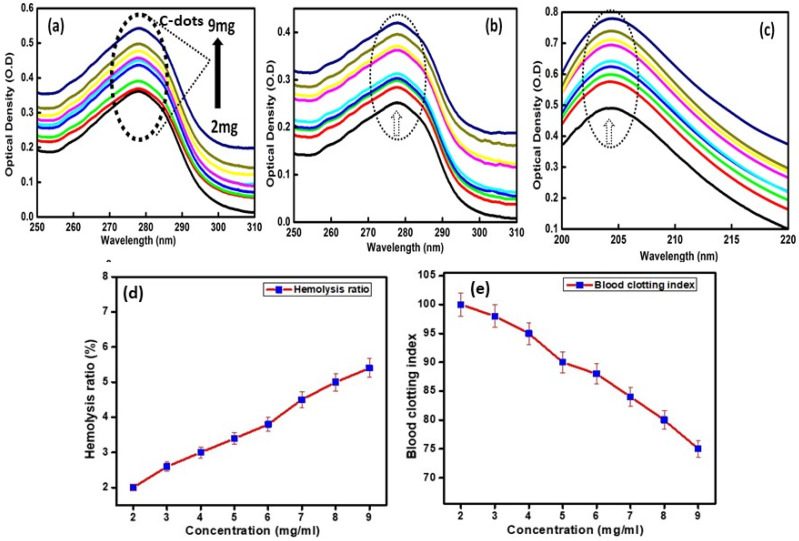
Effect of different concentrations (2 to 9 mg) of C-dots, ranging from 2 to 9 mg, on (**a**) BSA, (**b**) HSA, and (**c**) glutamic acid, and (**d**) hemolysis and (**e**) blood clotting indices (with <5% error bars).

The interaction behavior of different proteins (BSA, has, and glutamic acid) with the prepared C-dots provided valuable information about their quenching and binding ability. Among the different spectroscopic methods, the current study utilized the sensitivity of UV-vis. spectroscopy to study the electrostatic interaction mechanism of proteins and the prepared C-dots. The spectra displayed characteristic peaks at 275, 280, and 205 nm for BSA, has, and glutamic acid (Figure 5). In the case of BSA and HSA, the obtained peaks were mainly associated with the presence of the n-π^*^ transition (Figure 5a,b), whereas in case of glutamic acid the π-π^*^ transition was mainly responsible for the absorption peak (Figure 5c). The orientation of the n-π^*^ transition in BSA and HSA is mainly due to the presence of phenylalanine, tyrosine, and tryptophan amino acids in their structure. Additionally, it was observed that adding 2 mg of C-dots (a lower amount) into the protein solution significantly enhanced the value of the optical density. However, the optical density upsurged linearly when increasing the amount from 2 mg to 9 mg. The augmentation in the absorbance values of protein molecules in the presence of C-dots arose due to the variation in the microenvironment of the protein molecules. There was no significant shift in the peak positions of the chosen proteins in the presence of C-dots. These results suggested stable ground-state-complex formation between the proteins and C-dots. The well-organized and closed structure of the peptide bonds in proteins changed to an insecurely and loosely bound scrambled structure, due to the absorption of C-dots on the surface of proteins. The obtained zeta potentials of −12.8, −18.7, and −6.39 eV for BSA, has, and glutamic acid suggested the electrical charge stability of the protein molecules (Figure S6). The negative values of zeta potential can be mainly explained due to the carboxyl and different amine groups present on amino acids. The negative charges of the proteins changed to positive, with values equal to 61.6, 55.4, and 31.5 eV after adding C-dots in aqueous solution. These results support the complete binding of protein molecules with C-dots.

#### 3.3.2. Hemolysis and Blood Clotting Index Value in the Presence of C-Dots

The biocongenial performance of the highly stable prepared C-dots was also evaluated by assesssing out the hemolysis and blood clotting index activity at different concentrations (2–9 mg/mL). The adapted in vitro stability activity will provide a new pathway for the use of the developed C-dots in biological applications. The concentration variation behavior of the C-dots will provide a significant influential effect on blood systems (Figure 5d). It was observed that value of the hemolysis ratio increased linearly upon increasing the concentration of C-dots. The lower hemolysis ratio suggested the lower destruction of erythrocyte cells in the blood in the presence of C-dots. In addition, the decrease in hemolysis was found to be 5% for the highest concentration of C-dots used. The obtained results were considered in accordance with the ISO standard for hemocompatibility values. The obtained results support blood compatibility in the presence of the developed C-dots via preventing the hemolysis rate from increasing. Additionally, blood clotting index activity was chosen as an additional important area of study to carry out before utilizing the developed material for biomedical applications, in which they will come directly into contact with the blood system (Figure 5e). The results suggested that the blood clotting index decreased upon increasing the concentration of C-dots. Therefore, the obtained results suggested that the blood clotting mechanism was not affected in the presence of C-dots.

#### 3.3.3. MTT Assay

**Figure 6:**
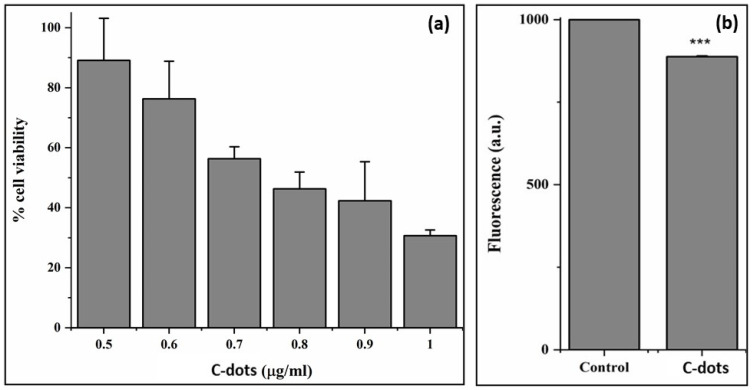
(**a**) Representation of MTT assay results for the developed C-dots at different concentrations and (**b**) ROS data obtained through fluorescence analysis (with <5% error bars). Statistical significance was assumed for *p*-values *** *p* < 0.05.

The effect of the C-dots on the growth of macrophages was studied by treating the cells with developed particles at different concentrations and incubating the cells for 24 h. The viability of cells was assessed using an MTT assay. The slight growth inhibition was obvious in the presence of C-dots. It was found that 1 µg/mL of the C-dots was able to decrease the growth of the tested cell lines close to 32% (Figure 6a). This is a very promising result regarding the concentration; according to National Cancer Institute, concentrations of any crude drug below 30 µg/mL are good enough to be considered as having cytotoxic effects [46]. The obtained results suggest that the developed C-dots were highly biocompatible and non-toxic at low concentration levels.

#### 3.3.4. ROS Detection Using DCFH-DA

As shown in Figure 6b, the control cells showed high fluorescence and these were compared to the cells treated with the C-dots. The increased fluorescence corresponded to an increased ROS level in the cells under study. In the present study, the level of oxidative stress was high in the control cells and when the developed C-dots were added to the cells, the fluorescence decreased, indicating the scavenging of the ROS molecules by the C-dots. The decrease in intensity was not very prominent in the cells treated with the developed C-dots. Rat hepatocytes treated with increasing concentrations of TiO_2_ and PLGA-PEO NPs also showed a decrease in the emission of DCFH-DA probe fluorescence [47], which correlated well with the current results.

### 3.4. Response Behavior of C-Dots towards Amino Acids

#### Selectivity Study of C-Dots

**Figure 7:**
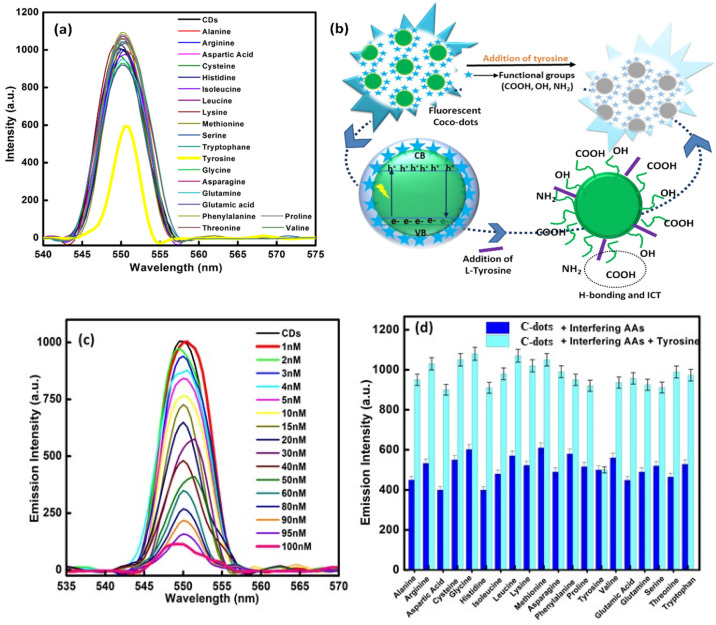

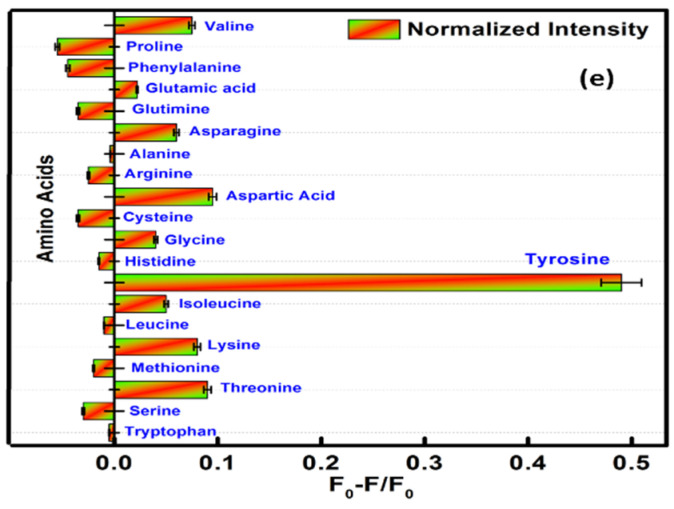
(**a**) Emission profile of C-dots in the presence of different amino acids, (**b**) mechanistic representation of the sensing of L-tyrosine amino acids using Coco C-dots, (**c**) concentration variation studies of L-tyrosine, (**d**) interference studies of C-dots and tyrosine in the presence of various other amino acids, and (**e**) normalized intensity of various amino acids along with tyrosine in the presence of C-dots (with <5% error bars).

Twenty amino acids (aspartic acid, serine, alanine, glycine, leucine, isoleucine, proline, valine, phenylalanine, tyrosine, tryptophan, histidine, glutamic acid, cysteine, asparagine, glutamine, lysine, arginine, methionine, and threonine) were utilized to certify the selectivity of the prepared C-dots (Figure 7a). Fluorescence spectroscopy was applied to evaluate the emission behavior of respective system, as it is considered as one of the most uncomplicated, inexpensive, and frequently used techniques in terms of sensing performance. The maximum excitation and emission wavelengths for the developed C-dots were kept at 295 nm and 530 nm during analysis. Under the optimum conditions, out of 20 different amino acids, the respective C-dots were found to be highly selective towards tyrosine. In case of tyrosine, the quenching (turn-off) phenomena was observed, whereas in the case of other amino acids, the emission intensity profile did not change much, which confirms the ultimate selectivity pattern of the developed C-dots towards tyrosine. Tyrosine is a non-essential amino acid and is considered one of the foremost targets for radical attacks. Tyrosine has been used by cells to produce proteins and continue the level of nutritional balance in the human body. It is therefore highly significant that the prepared C-dots sensed this amino acid and thus they hold potential in relation to the pharmaceutical and biotechnological industries due to their biocompatible and non-toxic nature. The current sensing method also demonstrated the detection of tyrosine at very low levels, representing a simple and low-cost methodology. The interior structure and resistance of the prepared C-dots is considered to be a potential feature related to the selectivity of particular amino acids. L-tyrosine is considered a neutral species and it has both anionic and cationic residues, i.e., (COO^−^) and (NH_3_^+^), respectively. The most important feature of tyrosine amino acid is the presence of a phenolic group which possess extreme charge transfer characteristics with the developed C-dots. The hydroxyl group of the phenol moiety causes a major charge transfer process, which suggests the outstanding sensitivity of tyrosine towards C-dots. Therefore, stable ionic complex formation occurred on both sides and was quite high in L-tyrosine. The hydrogen bonding between the different functional groups present on the exterior surface of the C-dots also suggested an interaction with L-tyrosine. The close proximity of hydrogen binding can lead to a quenching effect on the fluorescence intensity of the C-dots (Figure 7b). The slight shift in the fluorescence peak position of the C-dots in the presence of L-tyrosine further suggested a binding interaction. Inter-hydrogen bonding led to a decrease in the π-π^*^ and n-π^*^ states, which caused quenching in the fluorescence intensity of the formed C-dots. This may be been related to the intermolecular charge transfer (ICT) scheme [48,49]. The present interaction further suppressed various mechanistic behaviors such intramolecular hydrogen bonding and non-radiative relaxation, along with rotation and vibration. Among the different functional groups, N has a very high tendency towards hydrogen bond acceptors due to the presence of a lone pair. Therefore, selective analysis of L-tyrosine was carried out using the prepared C-dots through H-bonding. To scrutinize the effect of different concentrations of L-tyrosine, titration studies were carried with a fluoro-metric scheme (Figure 7c). The concentration was varied from 100 nM to 1 nM. It was observed that upon decreasing the concentration of tyrosine, the emission intensity increases linearly and after reaching a concentration of 1 nM, the emission intensity of L-tyrosine matches the intensity of the C-dots. It can thus be inferred that 1 nM was the minimum detectable concentration of L-tyrosine with the prepared C-dots under optimum conditions at room temperature. The obtained results reflect the potential of the developed C-dots to indicate the presence of tyrosine in an extremely uncomplicated manner. Further individual experiments were performed with the amino acids to investigate their individual nature, to ensure the selectivity of the sensed amino acid (L-tyrosine) and this was mixed with other chosen amino acids in the presence of C-dots (Figure 7d).

We interpreted the data to suggest that the emission profile of the developed C-dots does not greatly reflect alterations in the presence of other amino acids. However, only L-tyrosine alters the emission profile of the C-dots, which further shows the selectivity and sensitivity of this prepared sensor without any cross-interference from other amino acids. Furthermore, to analyze the competence and discriminatory aptitude of the developed system, similar experiments were carried out in the presence of various other types of inorganic anions (Cl^−^, Br^−^, OH^−^, NO_2_^−^, NO_3_^−^, SO_4_^2−^, CO_3_^2−^, AcO^−^, S_2_O_3_^2−^, and SO_3_^2−^) and biomolecules (urea, sucrose, glucose, fructose, ascorbic acid, tetracycline, and uric acid). The interfering mechanism was considered active only when the analytical discrepancy was >30% as compared to the signal obtained during the proposed analysis due to the absence of interfering ions. However, the obtained interfering results indicated that no additional interference was obtained for the analyte in the presence of different ionic species (Figure S7). Furthermore, the non-toxic and chemically stable nature of the developed C-dots makes them one of the best sensing precursors for the detection of L-tyrosine at room-temperature conditions. Furthermore, the normalized intensity plot of the developed C-dots was evaluated in the presence of various amino acids. The results supported the ultimate selectivity of L-tyrosine (Figure 7e). The developed sensor thus demonstrated excellent analytical performance, suggesting their potential use in biological applications. A concentration variation study of L-tyrosine was utilized in calculating the different analytical parameters, such as the detection limit and binding constant from a Stern–Volmer plot (F/F_0_ vs. conc. of tyrosine) and Benesi–Hildebrand plot (1/F − F_0_ vs. 1/conc. of tyrosine) (Figure S8). The values of the detection limit (D.L), quantitation limit (Q.L), and constant of binding (C.B) are reported in Table S3. The performance of the developed sensor is compared with those already reported in the literature in Table S4.

### 3.5. Practical Application of the Developed Sensor

**Figure 8:**
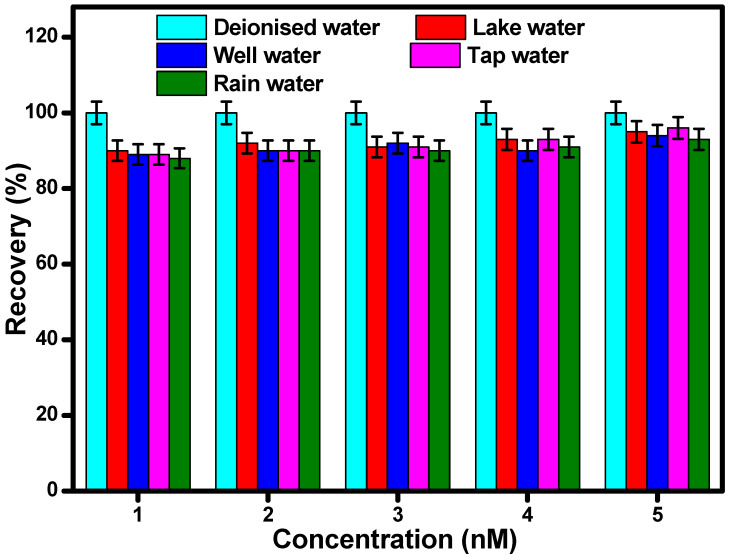
Recovery studies of L-tyrosine with C-dots in the presence of different water samples (with <5% error bars).

The developed C-dots reflected ultimate selectivity and sensitivity towards L-tyrosine. Therefore, its practical analytical performance was evaluated in the presence of real water samples taken from four different sources viz. lake water, rainwater, tap water, and well water (Figure 8). The respective water samples were spiked with different concentrations of L-tyrosine (1, 2, 3, 4, and 5 nM) using the standard addition method. The analytical performance studies were carried out in triplicate to investigate the reproducibility of the developed system. The recovery of each concentration varied from 92% to 95% in each water medium. The obtained fluorescence results indicated that the developed C-dots were selective, precise, and highly reliable, reflecting high sensitivity and good reproducibility in the presence of real water samples. The developed approach demonstrated the formation of a practical and accurate method for the quantitative detection of L-tyrosine in different water mediums.

## 4. Conclusions

In this paper, fluorescent C-dots were prepared usingcoconut coir waste as a precursor material. The developed scheme for the synthesis of C-dots can be carried out easily and reflects excellent selectivity and stability. Different analytical methods were employed to analyze the characteristic behavior of the C-dots. It was observed that the developed C-dots were completely synthesized in the nonorange with available functional groups. Furthermore, various biological activities were assessed in the developed C-dots to evaluate their biocompatible and non-toxic behavior. The cell viability of the developed C-dots was investigated using an MTT assay. The obtained results suggest the non-toxic behavior of the C-dots. The developed tyrosine-sensing ability leads to miniaturization of highly expensive instrumentation. The developed scheme was successfully utilized and applied for the detection of tyrosine among various amino acids in different water samples with satisfactory recovery values. The value of the detection limit was found to be 0.96 nM. From these results, it can be concluded that the developed C-dots can be utilized in the field of biological component sensing, with appropriate selectivity and sensitivity at low concentration levels.

## Data Availability

The data will made available on request.

## Supplementary Materials

The following supporting information can be downloaded at: https://www.mdpi.com/article/10.3390/nano12010162/s1, Figure S1. Excitation study of developed (a) CD_1_, (b) CD_2_ and (c) CD_3_ in wavelength range 200 to 650 nm by using fluorescence spectroscopy; Figure S2. Size distribution histogram of developed (a) CD_1_, (b) CD_2_ and (c) CD_3_ from HRTEM images by utilizing ImageJ software; Figure S3. EDX spectrum of developed CD_3_; Figure S4. TGA spectrum (a–c) and DSC spectrum (ai–ci) of developed Coco-dots at three different temperature condition i.e., 200, 250 and 300 °C; Figure S5. UV-visible absorption spectrum of developed C-dots in different pH media (pH = 2–13); Figure S6. Zeta potential distribution of (a) BSA, (b) HSA, (c) glutamic acid and (d–f) in presence of Coco-dots prepared at 300 °C; Figure S7. (a) Interference study of tyrosine in presence of anions and (b) biomolecules by using agarose derived CD’s; Figure S8. (a) Stern-volmer plot and (b) B-H plot of CD3 at different concentration level of tyrosine amino acid in presence of C-dots by using fluorescence data; Scheme S1. Schematic representation showing the synthesis mechansim of C-dots from coconut waste; Table S1. Representation of quantum yield value for developed C-dots by using tyrosine and tryptophan as a standard reference material; Table S2. Representation of different parameters evaluated out from TGA and DSC analysis; Table S3. Representation of different parameters evaluated from s-v plot and B-H plot; Table S4. Comparison of prepared C-dots with the previously reported C-dots from waste material. References [50,51,52,53,54,55,56] are cited in the supplementary materials.
