# Supplementary material for: Coconut Carbon Dots: Progressive Large-Scale Synthesis, Detailed Biological Activities and Smart Sensing Aptitudes towards Tyrosine
Source: Nanomaterials (Basel). 2022 Jan 3;12(1):162. doi: 10.3390/nano12010162 (PMC8746512; doi:10.3390/nano12010162)
Supplement: Supplementary file 1 [file nanomaterials-12-00162-s001.zip › nanomaterials-1515351-supplementary.pdf]

nanomaterials

Coconut Carbon Dots: Progressive Large-Scale Synthesis, Detailed Biological Activities and Smart Sensing Aptitudes towards Tyrosine

Pooja Chauhan [1], Deepa Mundekkad [2], Amitava Mukherjee [2], Savita Chaudhary [1,*], Ahmad Umar [3,4,*] and Sotirios Baskoutas [5,*]

[1] Department of Chemistry and Centre of Advanced Studies in Chemistry, Panjab University, Chandigarh 160014, India; pujachauhan.05@gmail.com
[2] Centre for Nanobiotechnology, Vellore Institute of Technology, Vellore 632014, India; deepamundekkad@gmail.com (D.M.); amit.mookerjea@gmail.com (A.M.)
[3] Department of Chemistry, College of Science and Arts, Najran University, Najran 11001, Saudi Arabia
[4] Promising Centre for Sensors and Electronic Devices (PCSED), Najran University, Najran 11001, Saudi Arabia
[5] Department of Materials Science, University of Patras, 265 04 Patras, Greece
* Correspondence: schaudhary@pu.ac.in (S.C.); ahmadumar786@gmail.com (A.U.); bask@upatras.gr (S.B.)

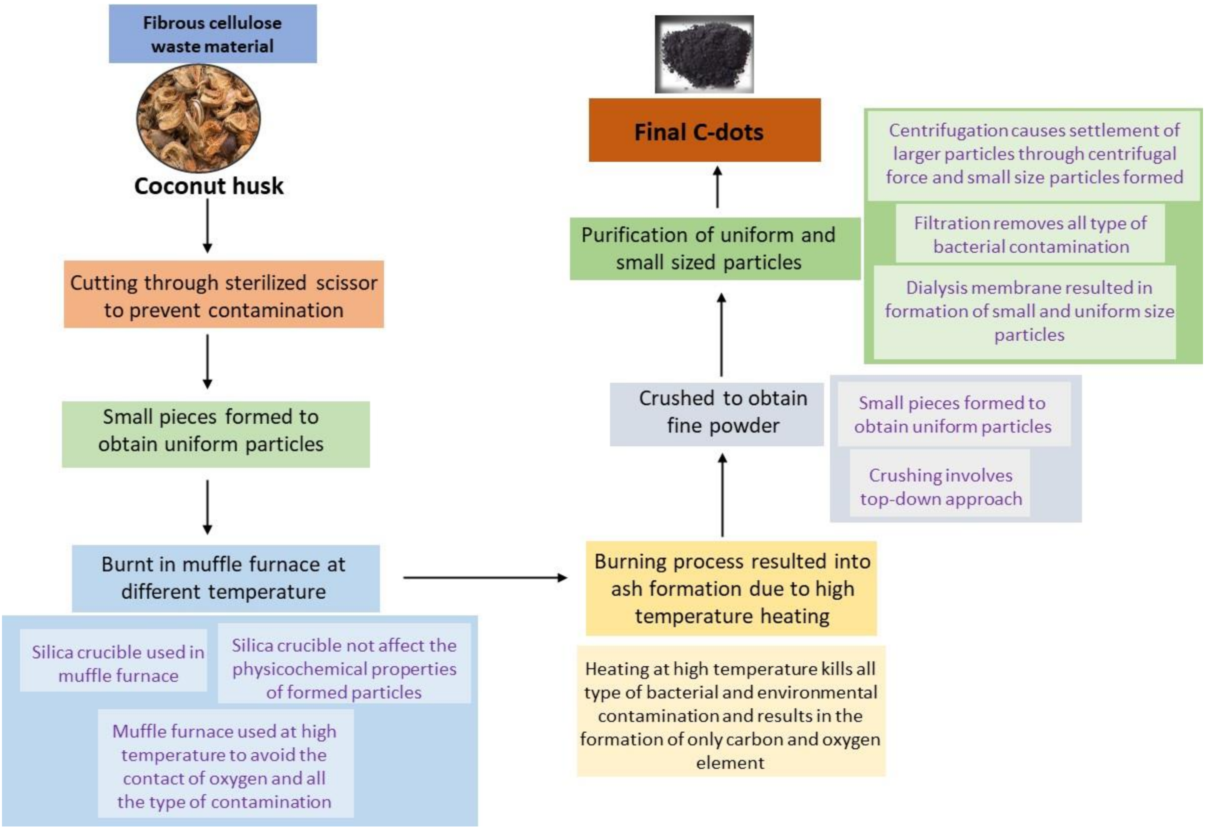

Scheme S1. Schematic representation showing the synthesis mechansim of C-dots from coconut waste.

Table S1. Representation of quantum yield value for developed C-dots by using tyrosine and tryptophan as a standard reference material.

S. No.	Carbon dot	Tyrosine as standard	Tryptophan as standard
1.	CD_1	32.4	31.1
2.	CD_2	35.7	35.6
3.	CD_3	38.6	39.5

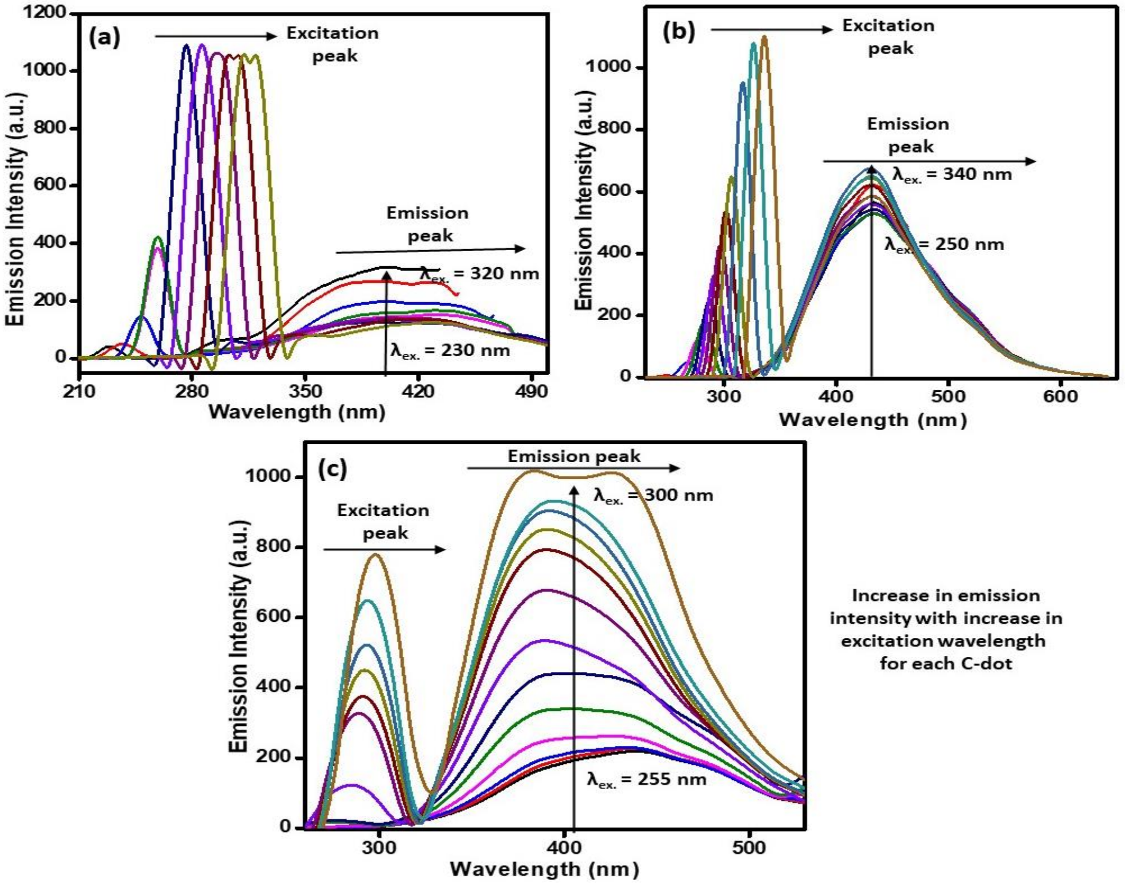

Figure S1. Excitation study of developed (**a**) CD_1, (**b**) CD_2 and (**c**) CD_3 in wavelength range 200 to 650 nm by using fluorescence spectroscopy.

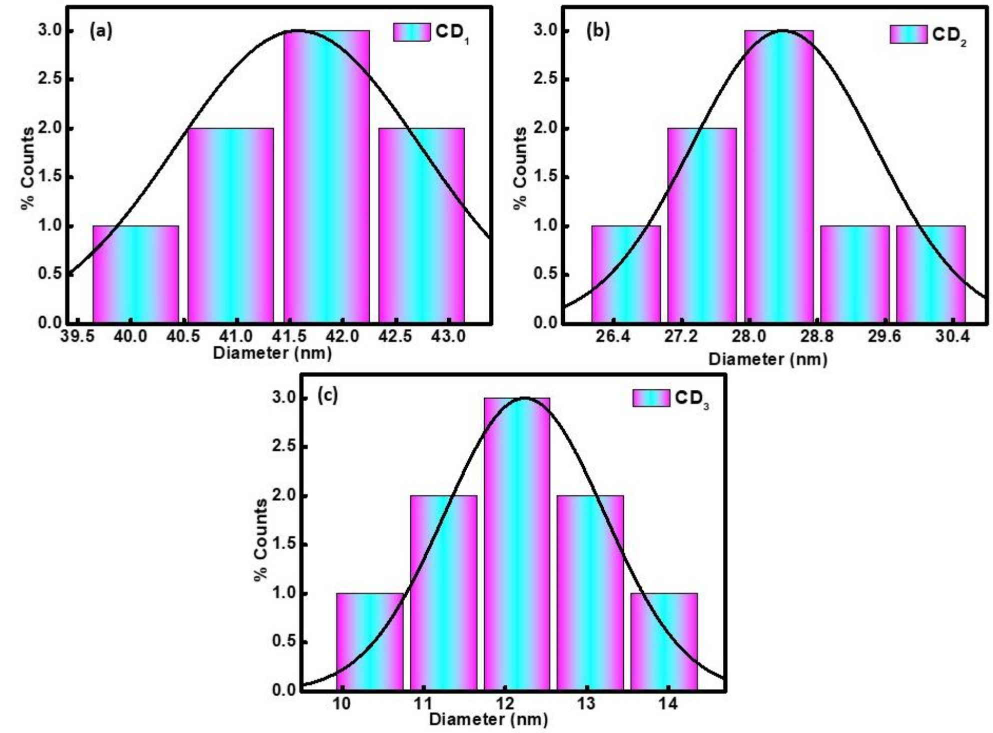

Figure S2. Size distribution histogram of developed (**a**) CD_1, (**b**) CD_2 and (**c**) CD_3 from HRTEM images by utilizing Image J software.

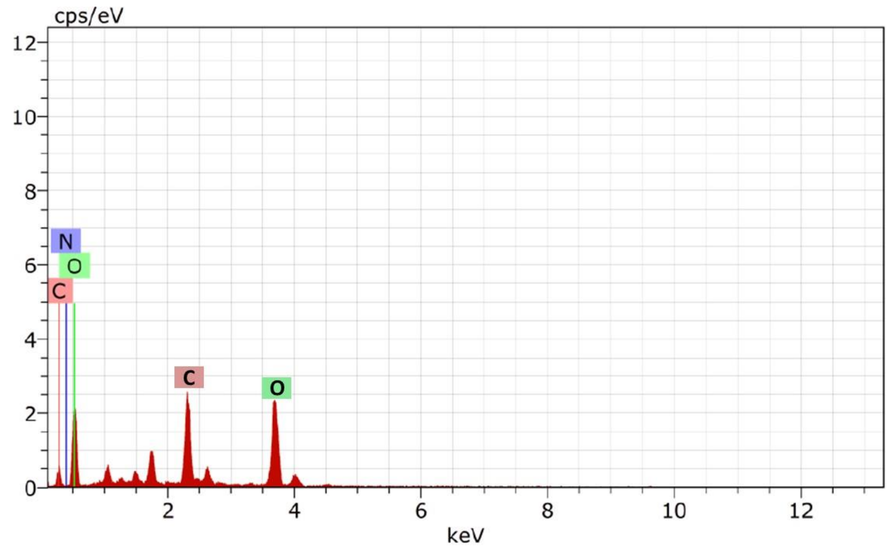

Figure S3. EDX spectrum of developed CD_3.

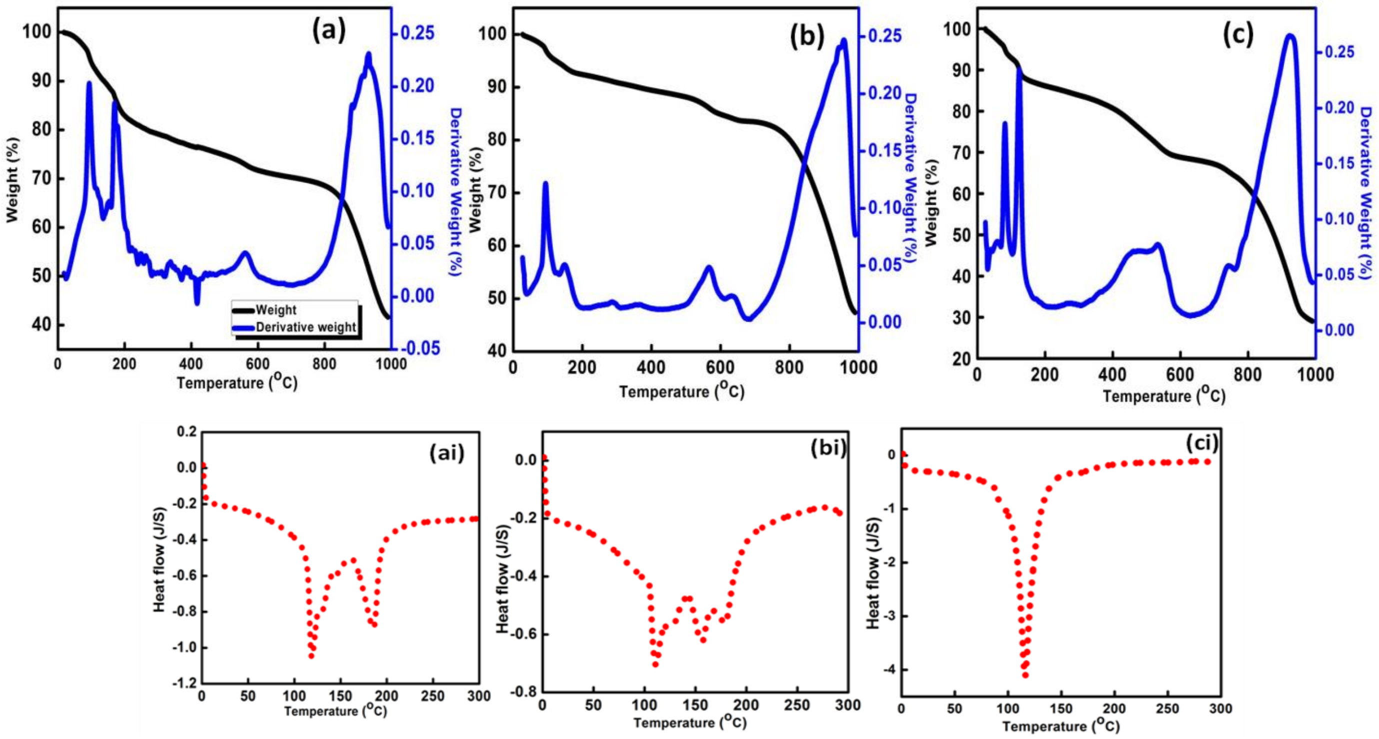

Figure S4. TGA spectrum (**a**–**c**) and DSC spectrum (**ai**–**ci**) of developed Coco-dots at three different temperature condition i.e., 200, 250 and 300 °C.

Table S2. Representation of different parameters evaluated out from TGA and DSC analysis.

Parameter	CD_1	CD_2	CD_3
		**TGA**	
**Weight loss temperature (℃)**	195 ℃, 598 ℃ and 985	198 ℃, 595 ℃ and 995	192 ℃, 593 ℃ and 987
**Weight loss (%)**	17.69 %, 29.48 % and 58.79 %	8.94 %, 15.54 % and 52.64 %	17.69 %, 18.28 % and 70.92 %
		**DSC**	
**T_o (℃)**	114.23, 168.42	105.32, 147.73	106.79
**T_g (℃)**	118.89, 186.06	110.78, 156.77	115.93
**T_m (℃)**	138.45, 205.89	128.35, 175.21	145.21
**ΔH (J/g)**	45.02, 35.43	15.33, 6.140	333.9

**ΔS**	0.32, 0.172	0.119, 0.035	2.299
**Peak**	2 exothermic	2 exothermic	exothermic

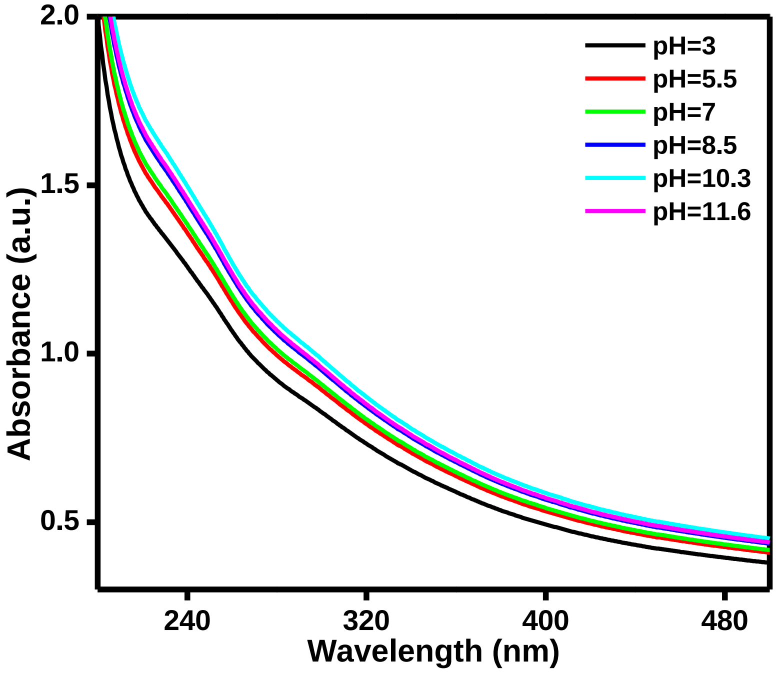

Figure S5. UV-visible absorption spectrum of developed C-dots in different pH media (pH = 2-13).

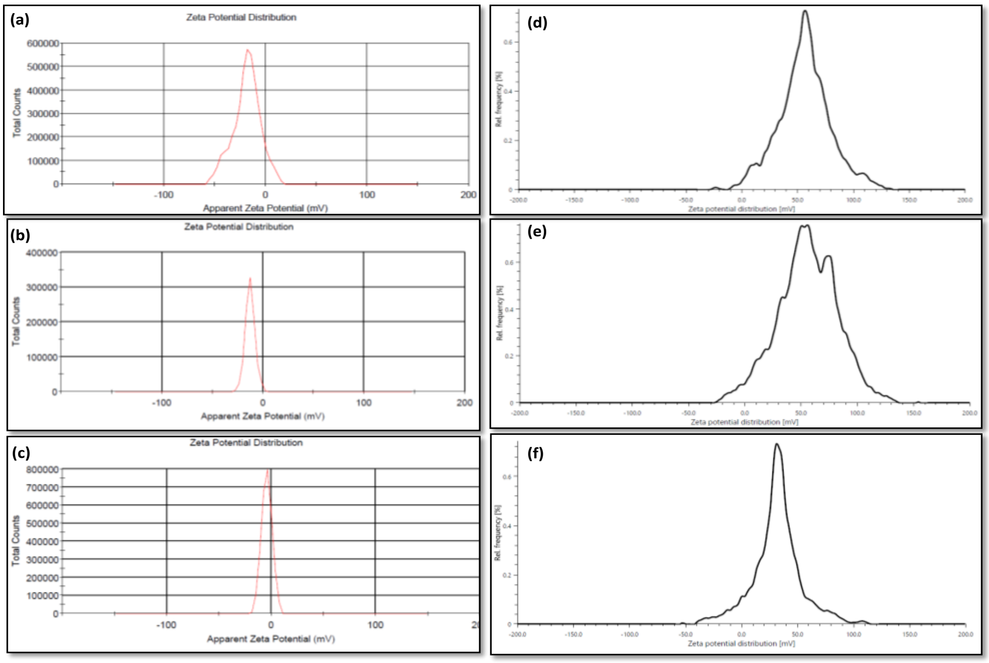

Figure S6. Zeta potential distribution of (**a**) BSA, (**b**) HSA, (**c**) glutamic acid and (**d**-**f**) in presence of Coco-dots prepared at 300 °C.

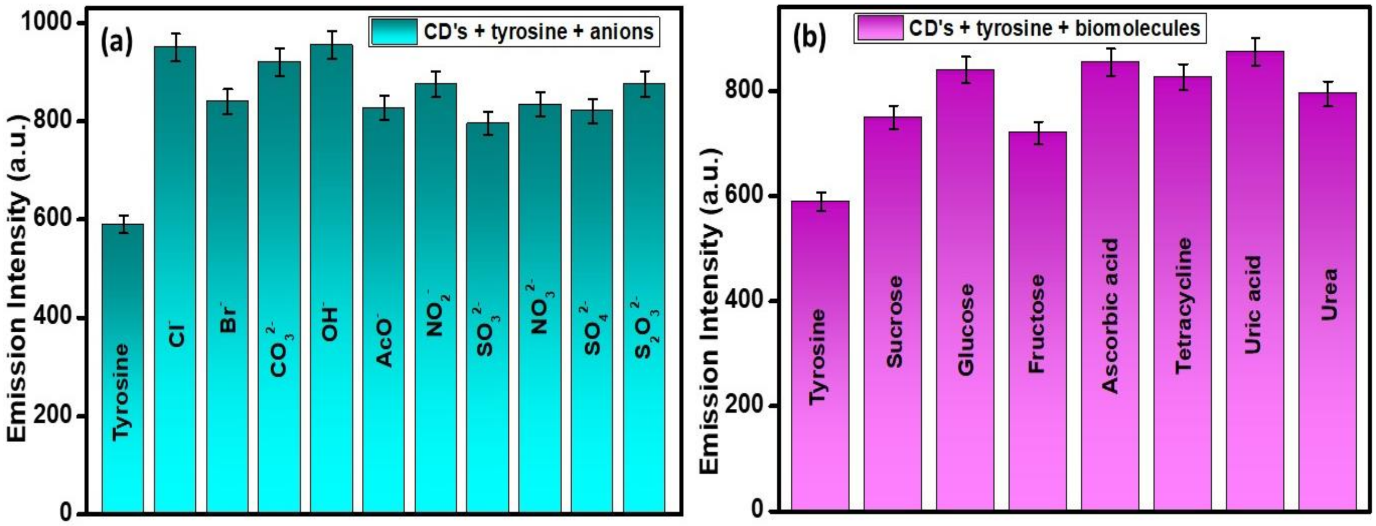

Figure S7. (**a**) Interference study of tyrosine in presence of anions and (**b**) biomolecules by using agarose derived CD's.

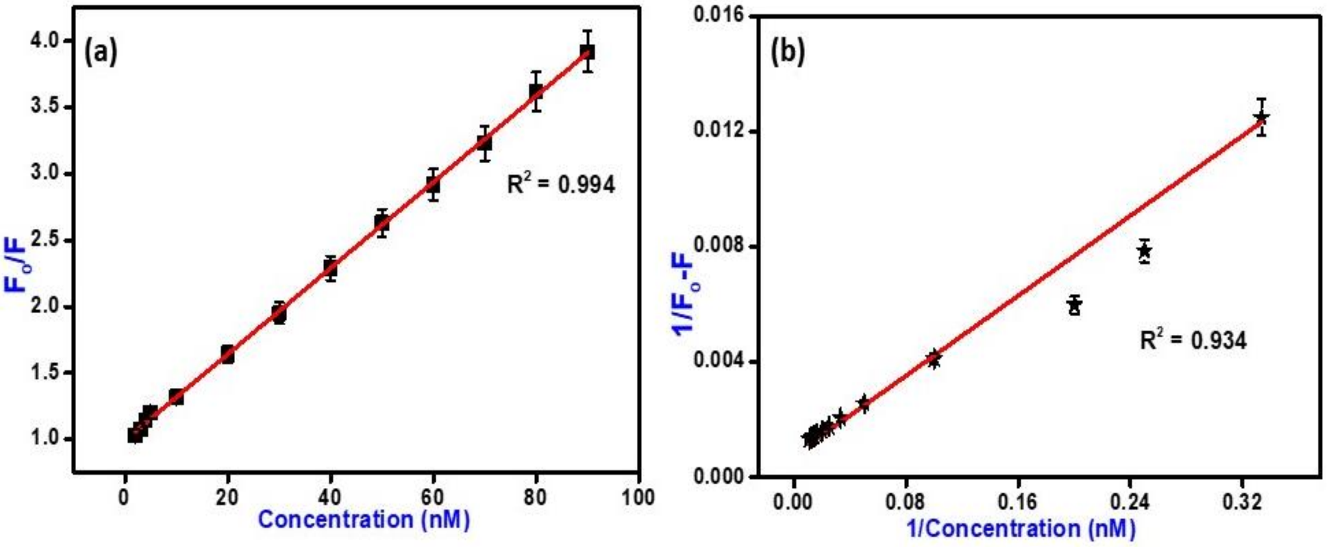

Figure S8. (**a**) Stern-volmer plot and (**b**) B-H plot of CD_3 at different concentration level of tyrosine amino acid in presence of C-dots by using fluorescence data.

Table S3. Representation of different parameters evaluated from s-v plot and B-H plot.

**1.**	**Intercept**	**Slope**	**R^2**	**D.L (nm)**	**Q.L (nm)**	Inter-cept	Slope	R^2	C.B
**2.**	0.993	0.0324	0.994	0.96	3.20	9.188	0.0310	0.97	296.38

Table S4. Comparison of prepared C-dots with the previously reported C-dots from waste material.

**S. No.**	**Precursor**	**Preparation method**	**Size**	**PL colour**	**Quantum yield (%)**	**Application**	**Ref.**
1.	leaves of Elettaria cardamomum	Ultra-sonication	<10 nm	blue	Not mentioned	Degradation of Congo red	[1]

2.	Whey, a diary waste	Thermal treatment	~4 nm	blue	11.4	Selenite sensing	[2]
3.	Waste paper	Solvothermal	2-4	blue	27	Anticounterfeiting and flexible display	[3]
4.	Waste carbon paper and cellulose	Hydrothermal	4.8	cyan	5.1	Detection of trinitrotoluene	[4]
5.	Wasted coffee grounds	Hydrothermal	2.5	yellow	Not mentioned	Anticounterfeiting	[5]
6.	Waste tobacco leaves	Hydrothermal	6.3	cyan	13.4	Sensing of tetracycline	[6]
7.	Straw mushroom	Hydrothermal	3-8	Red	Not mentioned	Adsorption of crystal violet and methylene blue	[7]
8.	Waste coconut husk	Hydrothermal	8	blue	54	Sensing of tyrosine	Present paper

References

1. Zaib, M.; Akhtar, A.; Maqsood, F.; Shahzadi, T. Green Synthesis of Carbon Dots and Their Application as Photocatalyst in Dye Degradation Studies. *Arab. J. Sci. Eng.* **2021**, *46*, 437–446, doi:10.1007/s13369-020-04904-w.
2. Devi, P.; Kaur, G.; Thakur, A.; Kaur, N.; Grewal, A.; Kumar, P. Waste derivitized blue luminescent carbon quantum dots for selenite sensing in water. *Talanta* **2017**, *170*, 49–55, doi:10.1016/j.talanta.2017.03.069.
3. Park, S.J.; Park, J.Y.; Chung, J.W.; Yang, H.K.; Moon, B.K.; Yi, S.S. Color tunable carbon quantum dots from wasted paper by different solvents for anti-counterfeiting and fluorescent flexible film. *Chem. Eng. J.* **2020**, *383*, 123200, doi:10.1016/j.cej.2019.123200.
4. Devi, S.; Gupta, R.K.; Paul, A.K.; Tyagi, S. Waste carbon paper derivatized Carbon Quantum Dots/(3-Aminopropyl)triethoxysilane based fluorescent probe for trinitrotoluene detection. *Mater. Res. Express* **2018**, *6*, 025605, doi:10.1088/2053-1591/aaf03c.
5. Hong, W.T., Park, J.Y., Chung, J.W., Yang, H.K. and Je, J.Y., 2021. Anti-counterfeiting application of fluorescent carbon dots derived from wasted coffee grounds. *Optik* **2021**, *241*, 166449, doi.org/10.1016/j.ijleo.2021.166449.
6. Liang, Y.-M.; Yang, H.; Zhou, B.; Chen, Y.; Yang, M.; Wei, K.-S.; Yan, X.-F.; Kang, C. Waste tobacco leaves derived carbon dots for tetracycline detection: Improving quantitative accuracy with the aid of chemometric model. *Anal. Chim. Acta* **2021**, *p.339269*, 339269, doi:10.1016/j.aca.2021.339269.
7. Zulfajri, M.; Kao, Y.-T.; Huang, G.G. Retrieve of residual waste of carbon dots derived from straw mushroom as a hydrochar for the removal of organic dyes from aqueous solutions. *Sustain. Chem. Pharm.* **2021**, *22*, 100469, doi:10.1016/j.scp.2021.100469
